# The efficacy of sentinel lymph node mapping with indocyanine green in cervical cancer

**DOI:** 10.1186/s12957-018-1341-6

**Published:** 2018-03-09

**Authors:** Ju-Hyun Kim, Dae-Yeon Kim, Dae-Shik Suh, Jong-Hyeok Kim, Yong-Man Kim, Young-Tak Kim, Joo-Hyun Nam

**Affiliations:** Department of Obstetrics and Gynecology, University of Ulsan College of Medicine, Asan Medical Center, 88 Olympic-ro, 43-gil, Songpa-gu, Seoul 05505 Republic of Korea

**Keywords:** Sentinel lymph node, Indocyanine green, Sensitivity and specificity, Detection, Uterine cervical neoplasms

## Abstract

**Background:**

Lymph node metastasis is a significant predictive factor for disease recurrence and survival in cervical cancer patients. Given the importance of lymph node metastasis, it is imperative that patients harboring metastasis are identified and can undergo appropriate treatment. Sentinel lymph node (SLN) mapping has drawn attention as a lymph node mapping technique. We evaluated the feasibility and efficacy of (SLN) mapping using indocyanine green (ICG) in cervical cancer.

**Methods:**

We performed a single-center, retrospective study of 103 surgically treated cervical cancer patients who underwent SLN mapping. After using ICG to detect SLN during surgery, we removed the SLNs followed by laparoscopic or robotic-assisted radical surgery and bilateral pelvic lymphadenectomy.

**Results:**

Stage IB1 was the most common (61.17%). At least one SLN was detected in all cases. Eighty-eight patients (85.44%) had bilateral pelvic SLNs. The mean number of SLN per patient was 2.34. The side-specific sensitivity was 71.43%, the specificity was 100%, the negative predictive value (NPV) was 93.98%, and the false negative rate (FNR) was 28.57%. In cases of tumors smaller than 2 cm with negative lymph node metastasis on imaging, the study revealed a side-specific sensitivity of 100%, a specificity of 100%, a NPV of 100%, and a FNR of 0%. Large tumor size (≥ 4 cm), a previous history of a loop electrosurgical excision procedure (LEEP), depth of invasion (≥ 50%), the microscopic parametrial (PM) invasion, and vaginal extension were significantly associated with the false-negative detection of SLN. Moreover, the microscopic PM invasion was the only risk factor of the false-negative detection of SLN in multivariate analysis.

**Conclusion:**

SLN mapping with ICG in cervical cancer is feasible and has high detection rate. The sensitivity of 100% was high enough to perform SLN biopsy alone in an early stage in which the tumor is less than 2 cm, with no lymphadenopathy on image examination. However, for large or invasive tumors, we would have to be cautious about performing SLN biopsy alone.

**Trial registration:**

Retrospectively registered 2017-0600.

## Background

According to the American Cancer Society, in 2016, an estimated 12,900 cervical cancer patients were newly diagnosed in the USA, and about 4100 people died of cervical cancer [1]. In Korea, there is a much higher incidence of cervical cancer compared with the West, although it has decreased gradually in the past few decades [2, 3]. Despite the fact that the present International Federation of Gynecology and Obstetrics (FIGO) staging system does not include lymph node metastasis in cervical cancer, lymph node metastasis is the most significant predictive factor for recurrence and survival in surgically treated cervical cancer patients [4–6]. It is known that approximately 20% of early cervical cancer patients have pelvic lymph node metastasis [7, 8]. If lymph node metastasis is found, the 5-year survival rate decreases from 92 to 64% [9, 10].

The standard surgical procedure for early cervical cancer includes pelvic lymphadenectomy [11]. The complications and morbidities arising from complete pelvic lymph node dissection, such as lymphocele and lymphedema, can be substantial problems in these patients [12]. Considering the status of lymph node metastasis, many patients may be undergoing unnecessary pelvic lymph node dissection and suffering from the resulting complications [13, 14]. Therefore, nodal assessment techniques, such as sentinel lymph node (SLN) mapping, are receiving much attention.

The SLN may be defined as the first regional lymph node or group of lymph nodes into which cancer cells are excreted from the primary tumor and invade during metastasis [15]. The detection of SLN in breast, penile, and skin cancer is a standard diagnostic procedure. If an SLN tests negative for cancer, full lymph node dissection can be omitted [16–18]. Several recent studies have proven the overall satisfactory efficacy of SLN biopsy in endometrial and cervical cancer [19, 20]. In early cervical cancer, the majority of studies of SLN biopsy have shown a detection rate of 95–100%, a false-negative rate (FNR) of 0–8%, and a negative predictive value (NPV) of 97–100% [21]. Despite these good results, there is still controversy because of the relatively high FNR in patients with large cervical cancers [11, 22–24].

In Korea, radical hysterectomy is preferred in the initial treatment of cervical cancer up to stages IB2 and IIA [25]. However, SLN biopsy is not widely used. In our center (Asan Medical Center, Korea), we have performed a retrospective review of SLN biopsy in cervical cancer. Our purpose was to determine the feasibility and efficacy of SLN mapping using indocyanine green (ICG) in patients with cervical cancer.

## Methods

### Study design and patient eligibility

We have performed a single-center, retrospective study of surgically treated cervical cancer patients with SLN mapping from August 2015 to January 2017 at Asan Medical Center, Korea. According to gynecologic oncologists’ judgments, patients over 18 years old who were pathologically diagnosed with cervical cancer and underwent surgical treatment were eligible for this study. International Federation of Gynecology and Obstetrics (FIGO) stage IA1 with lymphovascular space invasion (LVSI) to IIA disease was included, and preoperative magnetic resonance imaging (MRI) and 18F-fluoro-deoxyglucose positron emission tomography/computed tomography (18F-FDG PET/CT) were performed in all patients to exclude distant metastasis. Moreover, patients with a history of pelvic radiation therapy were excluded. Study approval was obtained from the institutional review board of Asan Medical Center, and all patients provided their informed consent. Study eligibility required surgery, including robotic-assisted or laparoscopic-assisted radical hysterectomy, trachelectomy with SLN mapping, and pelvic lymph node dissection. Five gynecologic oncologists performed the SLN mapping.

### Indocyanine green injection and surgical procedure

We selected ICG for SLN evaluation. A 25-mg single vial was used at each surgery. ICG was diluted with 20 ml of normal saline. Therefore, 1.25 mg/ml of ICG was used. After general anesthesia and just before placing the uterine manipulator, a total of 4 ml of ICG was injected directly to the right and left of the uterine cervix, both superficially and deep into the stroma. A fluorescence camera was used to identify the SLN with the retroperitoneum assessed. Only the SLN mapping technique was used. After the detection of SLNs, we removed and submitted them for frozen biopsy in case of suspicious metastasis. Followed by radical surgery, complete bilateral pelvic lymphadenectomy was performed in all patients. Moreover, some surgeons performed paraaortic lymph node dissection to the inferior mesenteric artery level if there was a metastasis of pelvic SLNs.

### Histopathology evaluation

Pathologists who specialized in gynecologic pathology examined all surgical specimens. The frozen biopsy was performed during operation. The result of SLN biopsy was obtained using ultrastaging, which was conducted in the same manner as for breast cancer [26]. Cytokeratin immunohistochemical staining was performed to check whether the lymph node was affected by metastasis. Hematoxylin-eosin staining was performed by dividing the specimen in half in case that it was not an SLN.

### Statistics

We collected all data from medical records. The descriptive statistics were used to analyze the demographic and clinical information and SLN mapping characteristics. Information such as sensitivity, specificity, NPV, and FNR was calculated as per patient and per hemipelvis to analyze the performance of SLN. We calculated the 95% confidence intervals for these rates using the exact binomial method.

We defined the false-negative detection of SLN as when the results of SLN biopsy was negative, but the result of none-SLN biopsy in the same side was positive for metastasis. The patients were divided into two groups depending on performance to find out the factors that influenced the false-negative detection of SLN. One group is a good performance group of no false-negative detection of SLN with bilaterality. Another group is a bad performance group of false-negative detection of SLN. And the two groups were analyzed using Fisher’s exact test. We also performed univariate and multivariate logistic regression of the risk factors affecting false-negative detection of SLN. For multivariable logistic regression, the stepwise variable selection was performed by backward elimination as final variables. All data were analyzed using SPSS software (version 21.0; SPSS Inc., Chicago, IL).

## Results

From August 2015 to January 2017, 103 cervical cancer patients were surgically treated with SLN mapping using ICG at Asan Medical Center. The demographic and clinical information of the patients is summarized in Table 1. The median patient age was 45 years (range, 29–77 years). The median body mass index was 22.42 kg/m^2^ (range, 17.99–29.89 kg/m^2^), and the median parity was 2 (42.7%, range 0–5). Stage IB1 disease was the most common (61.17%), followed by stage IB2 (19.42%). Forty-eight patients (46.60%) had undergone previous LEEP. The most common surgery was a laparoscopic radical hysterectomy (45.73%) followed by robotic-assisted radical hysterectomy (39.81%). Eight patients (7.77%) underwent fertility-sparing surgery.

**Table 1:** Demographic and clinical information (*n* = 103)

Characteristics	Values	Percentage (%)
Age (years)		
Median (range)	45 (29–77)	
Body mass index (kg/m^2^)		
Median (range)	22.42 (17.99–29.89)	
Parity (*n*)		
0	20	19.42
1	17	16.5
2	44	42.72
3	17	16.5
≥ 4	5	4.85
Surgical procedure (*n*)		
RARH	41	39.81
RAMRH	3	2.91
RART	1	0.97
LARH	47	45.73
LAMRH	4	3.88
LART	7	6.8
Previous LEEP history (*n*)		
No	55	53.4
Yes	48	46.6
HPV infection (*n*)		
No	6	5.82
Yes	65	63.11
Unknown	32	31.07
FIGO stage (*n*)		
IA1	8	7.77
IA2	6	5.83
IB1	63	61.17
IB2	20	19.42
IIA	6	5.83

*LEEP* loop electrosurgical excision procedure, *HPV* human papillomavirus, *FIGO* International Federation of Obstetrics and Gynecology, *RARH* robotic-assisted radical hysterectomy, *RAMRH* robotic-assisted modified radical hysterectomy, *RART* robotic-assisted radical trachelectomy, *LARH* laparoscopic-assisted radical hysterectomy, *LAMRH* laparoscopic-assisted modified radical hysterectomy, *LART* laparoscopic-assisted radical trachelectomy

The pathologic information is shown in Table 2. The most common histologic type was squamous cell carcinoma (*n* = 72, 69.90%), followed by adenocarcinoma (*n* = 23, 22.33%). The median tumor size based on the pathologic report was 2.4 cm (range, 0.1–8 cm). Twenty-five patients (24.27%) had large tumors of more than 4 cm, and 35 patients (33.98%) had LVSI. One patient had resection margin involvement. Moreover, the tumors of 11 patients (10.68%) extended into the vagina. The DOI of 55 patients (53.40%) was more than 50%. Nineteen patients (18.45%) had PM invasion, and 27 patients (26.2%) had nodal metastasis after lymphadenectomy on the final pathologic report.

**Table 2:** Pathologic information (*n* = 103)

Characteristics	Values	Percentage (%)
Histologic type (*n*)		
Squamous cell carcinoma	72	69.9
Endometrioid adenocarcinoma	1	0.97
Adenocarcinoma	23	22.33
Adenosquamous cell carcinoma	4	3.88
Other types	3	2.91
Grade (*n*)		
Well differentiated	9	8.74
Moderately differentiated	72	69.9
Poorly differentiated	15	14.56
Unknown	7	6.8
Tumor size (cm)		
Median (range)	2.4 (0.1–8)	
(*n*)		
< 4 cm	78	75.73
≥ 4 cm	25	24.27
Presence of LVSI (*n*)		
No	68	66.02
Yes	35	33.98
Resection margin involvement (*n*)		
No	102	99.03
Yes	1	0.97
Vaginal extension (*n*)		
No	92	89.32
Yes	11	10.68
Depth of invasion (*n*)		
< ½	48	46.6
≥ ½	55	53.4
Parametrial invasion (*n*)		
No	84	81.55
Yes	19	18.45
Lymph node metastasis		
No	76	73.79
Yes	27	26.21

*LVSI* lymphovascular invasion

Table 3 shows the characteristics of SLN mapping. At least 1 SLN was detected in all cases. Eighty-eight patients (85.44%) had bilateral pelvic SLNs. The mean number of SLNs per patient was 2.34. The most common SLN was the obturator lymph node (54.36%) followed by the external iliac lymph node (34.85%). One patient had a para-aortic SLN. The SLN was the only positive node in 10 patients (9.7%) of total patients. Four patients had lymph node metastasis on one side that were not detected as an SLN.

**Table 3:** SLN mapping characteristics (*n* = 103, SLN *n* = 241)

Variable	Value	%
Mean number of SLN removed per patient (*n*)	2.34	
SLN detention rate (*n*)		
Overall	103	100
Bilateral	88	85.44
Not mapping	0	0
SLN location (*n*)		
External iliac	84	34.85
Obturator	131	54.36
Common iliac	6	2.49
Internal iliac	9	3.73
Parametrial	9	3.73
Paraaortic	1	0.41
Prescral	1	0.41

*SLN* sentinel lymph node mapping

Calculated on a per patient basis, the sensitivity of SLN biopsy was 76.92% (95% CI 57.95–88.97%), the sensitivity was 100% (95% CI 95.00–100%), the FNR was 23.08%, and the NPV was 92.41% (95% CI 84.40–96.47%).

At least one SLN was detected in 191 out of 206 hemipelvises; the side-specific SLN detection rate was 92.72%. A total of 35 hemipelvises had lymph node metastasis, 25 of which involved the SLN, resulting in a sensitivity of 71.43% (95% CI 54.95–83.67%) and an FNR of 28.57%. One hundred and fifty-six hemipelvises had negative lymph node metastasis, and all of them had negative SLNs, resulting in a specificity of 100% (95% CI 97.60–100%) and an NPV of 93.98% (95% CI 89.27–96.70%). For tumors less than 2 cm with no lymphadenopathy on imaging tests, the study revealed a sensitivity of 100% (95% CI 20.65–100%), a specificity of 100% (95% CI 94.42–100%), and an NPV of 100% (95% CI 94.42–100%) in terms of side-specificity (Table 4).

**Table 4:** Performance of SLN mapping defined as side-specific

	All, *n* = 103, hemipelvis *n* = 191	*n* = 36, hemipelvis *n* = 66 Tumor size < 2 cm, negative lymph node metastasis on MRI and 18F-FDG PET/CT
Side-specific detection rate (%)	92.72	91.70%
Sensitivity (%, 95% CI)	71.43 (54.95–83.67)	100 (20.65–100)
Specificity (%,95% CI)	100 (97.60–100)	100 (94.42–100)
FNR (%)	28.57	0
FPR (%)	0	0
NPV (%, 95% CI)	93.98 (89.27–96.70)	100 (94.42–100)
Accuracy (%)	94.76	100

*SLN* sentinel lymph node mapping, *FNR* false-negative rate, *FPR* false-positive rate, *NPV* negative predictive value

False-negative detection of SLN occurred in nine patients. The patients were divided into two groups depending on their performance. Tumor size (*P* = 0.0019), microscopic PM invasion (*P* < 0.0001), depth of invasion (DOI) (*P* = 0.003), a previous history of a loop electrosurgical excision procedure (LEEP) (*P* = 0.0339), and vaginal extension status (*P* = 0.0415) were associated with false-negative detection of SLN using Fisher’s exact test (Table 5).

**Table 5:** Factors associated with false-negative detection of SLN

Factors	Group 1, *n* (%)	Group 2, *n* (%)	*P* value
Tumor size			
< 4 cm	65 (80.25)	3 (33.33)	0.0019
≥ 4 cm	16 (19.75)	6 (66.67)	
Previous LEEP			
No	39 (48.15)	1 (11.11)	0.0339
Yes	42 (51.85)	8 (88.89)	
Parametrium invasion			
No	72 (88.89)	3 (33.33)	< 0.0001
Yes	9 (11.11)	6 (66.67)	
Depth of invasion			
< 50%	42(51.85)	0(0)	0.003
≥ 50%	39(48.15)	9(100)	
Vagina extension			
No	73 (90.12)	6 (66.67)	0.0415
Yes	8 (9.88)	3 (33.33)	
Resention margin involvement			
NO	80 (98.77)	9 (100)	> 0.999
Yes	1 (1.23)	0	
FIGO staging			
IA1	6 (7.41)	0	0.5493
IA2	6 (7.41)	0	
IB1	51 (62.96)	5 (55.56)	
IB2	13 (14.28)	3 (33.33)	
IIA	5 (6.17)	1 (11.11)	

*SLN* sentinel lymph node mapping; *LEEP* loop electrosurgical excision procedure; *FIGO* International Federation of Obstetrics and Gynecology; *Group 1*, no false-negative detection group of sentinel lymph node with bilateral detection of sentinel lymph node; *Group 2*, false-negative detection group of sentinel lymph node

Also, we performed univariable logistic regression of the risk factors affecting false-negative detection of SLN, and the results are shown in Table 6. The results of the univariate analysis revealed that the risk factors were tumor size of more than 4 cm (odds ratio (OR) 8.125, 95% confidence interval (CI) 1.831–36.049; *P* = 0.0059), LVSI (OR 8.674, 95% CI 1.675–44.911; *P* = 0.01), and microscopic PM invasion (OR 16.001, 95% CI 3.398–75.35; *P* = 0.0005). For the multivariate analysis, the variable selection was performed by backward elimination as final variables. Finally, the microscopic PM invasion was the only selected one as a final variable for the false-negative detection of SLN.

**Table 6:** Logistic regression for risk factors affecting false-negative detection of SLN

Characteristics	Univariate			Stepwise multivariate analysis		
	OR	95% CI	*P*	OR	95% CI	*P*
No previous LEEP	0.135	0.016–1.126	0.0643			
Size ≥ 4 cm	8.125	1.831–36.049	0.0059			
LVSI present	8.674	1.675–44.911	0.01			
Pathological parametrial invasion	16.001	3.398–75.35	0.0005	16.001	3.398–75.35	0.0005
Vaginal extension	4.563	0.953–21.851	0.0575			
Depth of invasion (cm)	1.132	1–3.444	0.0504			
FIGO stage	1.726	0.822–3.623	0.1494			

*SLN* sentinel lymph node, *LEEP* loop electrosurgical excision procedure, *LVSI* lymphovascular space invasion, *FIGO* International Federation of Obstetrics and Gynecology

## Discussion

Our study was conducted on 103 cervical cancer patients who underwent SLN biopsy using ICG. The detection rate of SLN using ICG was 100%, and the bilateral detection rate was 85.44%. Our study showed improved detection rates over the previous traditional radiocolloid/blue dye mapping procedure [27]. The ICG mapping technique has more advantages, including avoidance of radiation exposure, no pain, and a much lower cost than other traditional techniques [27]. According to a study of SLN mapping with ICG by Beavis et al., at least 1 SLN was detected in all cases and the bilateral detection rate of SLN was 87% [28]. Similarly, we showed the feasibility of using ICG and its high detection rate.

In all cases, the sensitivity per hemipelvis was 71.43% (95% CI 54.95–83.67%), while the FNR was 28.57%. There were nine cases of false-negative SLN detection. These are much less sensitive than the rates reported in a recent meta-analysis study and the SENTOCOL study [20, 29]. Our patients had more advanced disease stages and larger tumors than these studies. Therefore, we additionally analyzed the factors affecting the false-negative detection of SLN.

Our study showed that risk factors for the FNR were a large tumor size (≥ 4 cm), LVSI, and microscopic PM invasion in univariate analysis, and only microscopic PM invasion was a significant factor among them by backward elimination for the multivariate analysis. Furthermore, we found that DOI, a previous history of a LEEP and vaginal extension status, were also associated factors for the false-negative detection of SLN. These factors may be associated with the destruction of the lymphatic channel. When injecting ICG into the cervix, it is sometimes difficult to find the injection site if the tumor is bulky. According to Zarganis et al., LVSI and a larger tumor size often affect false-negative detection of SLN and may increase the risk thereof [30]. Salvo et al. also explained that debris or tumor emboli from larger tumors clogging lymphatics might keep mapping substances from reaching nodes [31]. According to Dargent et al.’s study of SNL mapping of cervical cancer patients with radiotracer/blue dye, the history of previous conization was associated with mapping failure [32].

On the other hand, for the cases in which the tumor was smaller than 2 cm, and where both 18F FDG PET/CT and MRI showed lymph node negativity, the sensitivity, specificity, and NPV were 100%. This value is similar to that in previous studies [20, 29]. Another recent retrospective study of SLN biopsy in early cervical cancer showed a high sensitivity of 96.4% (95% CI 79.8–99.8%) and an NPV of 99.3% (95% CI 95.6–100%) [31]. Given our results, it seems that detailed criteria are required for SLN biopsy in early cervical cancer. For example, specifically, the tumor should be less than 2 cm diameter, and there should be no suspicious lymphadenopathy on imaging tests for best results with SLN mapping with ICG.

The strength of our study is that there were a large number of cases that could be operated from a large tumor size up to stage IIA. And our study is the only one that makes the comparison of the risk factors between the group with false-negative detection of SLN using ICG and that having no false-negative detection of SLN using ICG.

One of the limitations of our study is that it is a retrospective study with a small sample size. We included all patients who underwent SLN biopsy with ICG during this period to minimize the selection bias.

## Conclusion

In conclusion, SLN mapping with ICG in cervical cancer is feasible and has high detection rate. And it may be possible to perform SLN biopsy alone in early-stage cervical cancer when the tumor is less than 2 cm and lymph node metastasis is not suspected on image examination**.** However, for large or invasive tumors, we would have to be cautious about performing SLN biopsy alone. Large-scale prospective studies are needed to determine whether SLN biopsy only can be performed in early cervical cancer. Furthermore, a detailed guideline for the SLN biopsy in cervical cancer will need to be established.

## Abbreviations

18F-FDG PET/CT: 18F-fluoro-deoxyglucose positron emission tomography/computed tomography
FIGO: International Federation of Gynecology and Obstetrics
FNR: False-negative rate
ICG: Indocyanine green
LEEP: Loop electrosurgical excisional procedure
LVSI: Lymphovascular space invasion
MRI: Magnetic resonance imaging
NPV: Negative predictive value
PM: Parametrial
SLN: Sentinel lymph node
